# The impact of mass drug administration of antibiotics on the gut microbiota of target populations

**DOI:** 10.1186/s40249-022-00999-5

**Published:** 2022-06-30

**Authors:** Ethan K. Gough

**Affiliations:** Department of International Health, Human Nutrition Program, Johns Hopkins Bloomberg School of Public Health, Baltimore, MD USA

**Keywords:** Antibiotics, Mass drug administration, Microbiota, Microbiome

## Abstract

Antibiotics have become a mainstay of healthcare in the past century due to their activity against pathogens. This manuscript reviews the impact of antibiotic use on the intestinal microbiota in the context of mass drug administration (MDA). The importance of the gut microbiota to human metabolism and physiology is now well established, and antibiotic exposure may impact host health via collateral effects on the microbiota and its functions. To gain further insight into how gut microbiota respond to antibiotic perturbation and the implications for public health, factors that influence the impact of antibiotic exposure on the microbiota, potential health outcomes of antibiotic-induced microbiota alterations, and strategies that have the potential to ameliorate these wider antibiotic-associated microbiota perturbations are also reviewed.

**Graphical Abstract:**

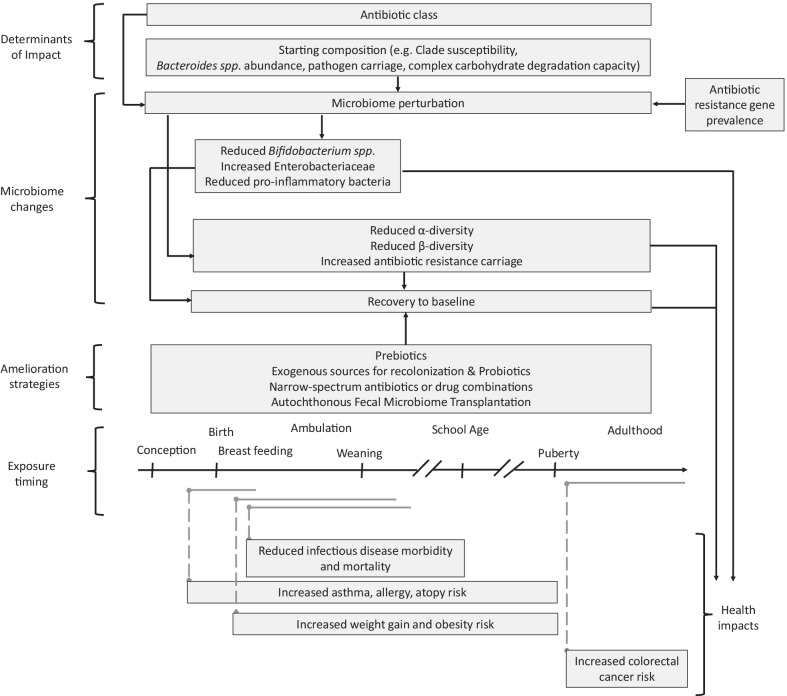

## Background

Greater accessibility to antimicrobials has contributed to an estimated 65% increase in human antibiotic consumption from 2000 to 2015 [1]. The clearest consequence is greater selection for antibiotic resistance, which has made common infections difficult to treat in recent years [2]. Antibiotic use can also have off-target effects, meaning that the concentrations at which antibiotics are administered elicit antimicrobial effects beyond elimination of pathogenic bacteria that impact the composition and function of the microbiome [3, 4]. Next-generation and targeted sequencing have demonstrated that antibiotics induce a reduction in gut bacterial biomass, taxonomic diversity, and functional diversity [5].

Virtually every niche of the human body harbors diverse microbial communities [6], even sites once thought as sterile, such as the bladder [7] and lungs [8]. The gastrointestinal tract is home to the greatest microbial biomass and biodiversity in the human body. Advances in DNA and RNA sequencing technology have revolutionized our ability to quantify and explore the dynamics of the gut microbiome, which elicits wide reaching effects on physiologic development, immune function, homeostasis, nutrient acquisition, and protection against infections [9, 10]. A causal role of the gut microbiome in development of celiac disease [11], inflammatory bowel disease [12], obesity [13, 14], undernutrition [15, 16], metabolic syndrome [17], asthma [18], and brain and nervous system development [19], have been clearly demonstrated using animal models. However, microbiome signatures associated with disease in clinical studies have been inconsistent across studies and outcomes [20]. Absence of clear evidence in human studies has even been demonstrated for disease conditions where the role of the microbiome is widely accepted, such as celiac disease [21] and IBD [22], due to heterogeneity in study designs [21], study procedures [21] or disease subtypes [22].

The prevailing view holds that microbial colonization of the gastrointestinal tract begins at birth in full-term infants, after which the microbiome progresses through a succession of identifiable shifts in composition [23]. Immediately after birth, the infant gut is predominantly colonized by facultative anaerobic Enterobacteriaceae [24]. Over the next few days, microbiota composition shifts to predominantly anaerobic bacteria [24]. In preterm neonates, however, gut colonization by anaerobes is delayed, likely due to delayed gut and immune system maturation, greater exposure to the hospital environment, and greater exposure to antibiotics [25].

The major initial sources of bacteria for colonization of the infant gut are the mother’s vaginal, fecal, and skin microbiota and the immediate environment [24, 26, 27]. In vaginally delivered newborns, the gut microbiota is more similar to the mothers’ vaginal microbiota [28]. The maternal gut microbiota is also transferred to the infant during vaginal delivery [29–31]. By contrast to vaginal delivery, cesarean delivered newborns’ gut microbiota more closely resembles the mothers’ skin and the environmental microbiota the infant is exposed to.

After birth, infant diet largely determines the composition of the gut microbiota. In breastfed infants, *Bifidobacterium* spp. are the most abundant bacteria [23, 32]. Human breast milk is rich in oligosaccharides, which *Bifidobacterium* spp. preferentially consume [33]. Weaning induces the next major shift in composition. A wider variety of nutrients in solid foods and reduced availability of milk oligosaccharides correspond with an increase in diversity and a decrease in Bifidobacterium. In the subsequent period, the infant microbiota develops a more adult-like composition, with greater bacterial diversity and evenness by 2–3 years of age [24, 26, 27, 34]. Composition and function may continue to develop through childhood and early adolescence [35, 36]. Geography also plays an important role. Infant microbiota composition differs between high-income versus low- and middle-income populations [23, 37, 38], likely driven by differences in diet, sanitation, and human genetics [25, 39]. Variations in the genetic potential for human lactase production, for example, explain Bifidobacterium abundance [40]. Diet and antibiotic exposure are also key factors that continue to influence this ongoing process [41, 42].

While the on-target effects of antibiotic exposure on pathogen clearance are beneficial to host health, the off-target effects of antibiotic exposure on gut microbiome composition and function can result in either detrimental or beneficial effects on host health, depending on the initial composition and function of the autochthonous microbial community. This is particularly relevant given recent increased interest in mass drug administration (MDA) with antibiotics—empirically administering drugs to an entire at-risk community or population to suppress pathogen growth—in view of, for example, global targets to reduce neonatal and under-5 mortality by 2030 [43], global targets for elimination of trachoma [44] and yaws [45], guidelines for cotrimoxazole prophylaxis among persons living with HIV [46], and recommendations for prevention of maternal and newborn peripartum infections by Group B Streptococcus (GBS) [47, 48]. Recent interest in MDA with antibiotics has also gained traction due to concerns about emergence of more difficult to treat enteric [49] and non-enteric [49, 50] infections and potential transmission of resistant pathogens to more vulnerable populations [49, 51]. The need to balance these risks with the demonstrated benefits of MDA with antibiotics [52, 53] is of particular relevance.

This narrative review describes studies that used high-throughput 16S rRNA gene amplicon sequencing or next generation whole genome shotgun sequencing (WGS) to investigate the impact of antibiotic use on gut microbiome composition and function in the *context of MDA*.

## Main text

### Methods

Data for this review were initially identified through a search of PubMed up to March 2021 using the search terms “mass drug administration”, “antibiotic*”, and “microbiome” or “microbiota”. This search provided an initial set of studies relevant to the topic. References from the initially identified studies were also searched and included in this review if relevant. Publications were included if they (i) reported the impact of antibiotic use on gut microbiome composition or function in the context of MDA and (ii) used high-throughput methods to survey the gut microbiome because those methods provide a more comprehensive catalogue of microbiome composition and antibiotic-associated alterations. Only articles published in English were included. The search period was not restricted. Included studies are summarized in Tables 1, 2, 3. To gain further insight into the findings of these MDA studies, other studies that investigated the factors which determine the impact of antibiotic use on the gut microbiome and its recovery, strategies to ameliorate these effects, and potential health outcomes are also discussed.

**Table 1:** Characteristics of studies included in this review of the impact of mass drug administration with antibiotics for trachoma, yaws elimination or child mortality reduction on the human gut microbiome

Reason for MDA	First author	Year	Study design	Specimen collection and Sequencing	Country	Target population	Intervention	Major findings
Trachoma and Yaws elimination, reduce child mortality	Doan, T [62]	2017	RCT	Rectal swab, 16S rRNA gene V3–V4 amplification and sequencing	Niger	1–60 month old children (*n* = 80)	Single dose oral azithromycin (20 mg/kg) once versus placebo	27%–31% decrease in microbiome diversity 5 days post-treatment
Trachoma and Yaws elimination, reduce child mortality	Parker, EPK [60]	2017	RCT	200 mg stool, 16S rRNA gene V4 amplification and sequencing	India	6–11 month old children (*n* = 120)	3-day course of oral azithromycin (administered once daily at a dose of 10 mg/kg) versus placebo	7% reduction in microbiome richness 12 days post-treatment, driven by reductions in abundance of *Akkermansia muciniphila* and Proteobacteria (enteropathogenic *E. coli*, enteroaggregative *E. coli*, enterotoxigenic *E. coli* and *Campylobacter sp.*)
Trachoma and Yaws elimination, reduce child mortality	Doan, T [55]	2018	Cluster RCT	Rectal swab, whole metagenome sequencing	Niger	1–60 month old children (*n* = 30 communities, 10 infants/community)	Single dose oral azithromycin (20 mg/kg body weight) given every 6 months versus placebo	16%–22% decrease in microbiome diversity after 12 months
Trachoma and Yaws elimination, reduce child mortality	Oldenburg, CE [61]	2018	RCT	Rectal swab, 16S rRNA gene V3–V4 amplification and sequencing	Burkina Faso	6–59 month old children (*n* = 124)	5-day course of either oral amoxicillin (25 mg/kg/d twice-daily doses), oral azithromycin (10 mg/kg dose on day 1 and then 5 mg/kg once daily for 4 days), oral cotrimoxazole (240 mg once daily), versus placebo	32% decrease in microbiome diversity with azithromycin at 5 days post-treatment
Trachoma and Yaws elimination, reduce child mortality	Doan, T [56]	2019	Cluster RCT	Rectal swab, whole metagenome sequencing	Niger	1–60 month old children (*n* = 30 communities, 10 infants/community)	Single dose oral azithromycin (20 mg/kg body weight) given every 6 months versus placebo	Reduced abundance of *Campylobacter upsaliensis* and *Campylobacter hominis*, and reduced abundance of bacterial metabolic pathways predominantly geared toward microbial survival, growth and inflammation after 24 months
Trachoma and Yaws elimination, reduce child mortality	Doan, T [59]	2020	Cluster RCT	Rectal swab, whole metagenome sequencing	Niger	1–60 month old children (*n* = 30 communities, 10 infants/community)	Single dose oral azithromycin (20 mg/kg body weight) given every 6 months versus placebo	7.5 times (95%CI: 3.8–23.1) greater abundance of bacterial genetic determinants for macrolide resistance, 3.59 (1.73–8.20) greater abundance of genetic determinants of metronidazole resistance, 1.98 (95%CI:1.10–4.57) greater abundance of genetic determinants of beta-lactam resistance, and 1.75 (95%CI:1.03–4.02) greater abundance of genetic determinants tetracycline resistance in the microbiome after 48 months
Trachoma and Yaws elimination, reduce child mortality	Hinterwirth, A [63]	2020	RCT	Rectal swab, whole metagenome sequencing	Burkina Faso	1–60 month old children (*n* = 62)	Single dose oral azithromycin (20 mg/kg) once versus placebo	Reduced *Campylobacter jejuni*, *Campylobacter ureolyticus*, and *Campylobacter hominis* 5 days post-treatment

### Impact of MDA on gut microbiota

#### Prophylaxis in the general population

Azithromycin is an integral part of the World Health Organization (WHO) strategy to eliminate trachoma [54] and yaws [45]. Five randomized controlled trials (RCT) investigated the impact on the gut microbiota of MDA with azithromycin for prophylaxis in the general population. The gut microbiome was characterized primarily in terms of α-diversity, which quantifies the number of different bacterial taxa in an individual microbiota and the uniformity of their abundances (Table 1). One RCT investigated impacts on the microbiota after repeated biannual treatment over a follow-up period of 48-months (long-term use), and four RCTs investigated impacts in the period immediately following a single dose or course of treatment (short-term use). All five RCTs were conducted in low- and middle-income countries (LMICs).

The impact of long-term use was reported in three studies representing data from a single cluster-RCT. MDA comprised of one dose of azithromycin given biannually to every child 1–60 months of age in participating communities in Niger. A 16%–22% decrease in microbiota α-diversity was reported after 12 months [55]. However, longer follow-up at 24 months found reduced abundance of *Campylobacter upsaliensis* and *Campylobacter hominis* with azithromycin treatment, as well as reduced abundance of bacterial metabolic pathways predominantly geared toward microbial survival, growth and inflammation, which may partly explain the reported benefits to child mortality [56]. *C. upsaliensis* is a well-recognized food-borne enteric pathogen in high-income settings, particularly among children in rural populations [57]; while *C. hominis* has been associated with gut inflammation and bacteremia [58]. By contrast, after 48 months of treatment, there was greater abundance of bacterial genetic determinants for macrolide resistance in the gut [59]. Azithromycin also selected for determinants of non-macrolide resistance, including beta-lactam, tetracycline and fluoroquinolone antibiotic resistance genes [59]. Other RCTs of MDA with azithromycin have not reported on changes in antibiotic resistance gene carriage by the fecal microbiome.

The impact of short-term use was reported in four RCTs, in three countries (Table 1). Two RCTs (India [60] and Burkina Faso [61]) allocated children up to 60 months of age to a single multi-day course of antibiotics. Azithromycin treatment reduced microbiota α-diversity by up to 32% relative to placebo. Reductions in *Akkermansia muciniphila* and Proteobacteria were also reported [60]. The latter group of bacteria includes common pathogens such as *E. coli* and *Campylobacter* spp. [60] Furthermore, acquisition of enteric infections by *E. coli* pathotypes and *Campylobacter* spp. during the 2 week study period was reduced, and clearance of baseline infections with these enteric pathogens was increased among azithromycin-treated infants, as confirmed by polymerase chain reaction (PCR). [60] Two RCTs of single dose azithromycin (Niger [62] and Burkina Faso [63]) conducted in a comparable age group, also found a 27%–31% reduction in α-diversity [62], or a decrease in *C. jejuni*, *C. ureolyticus*, and *C. hominis* with azithromycin treatment [63]. However, α-diversity in children treated with amoxicillin or cotrimoxazole was not significantly altered [61].

Together, these studies show a clear reduction in α-diversity caused by azithromycin alongside a reduction in pathogens of the Enterobacteriaceae family. MDA trials of amoxicillin or cotrimoxazole did not report any such effects.

#### Prophylaxis in persons-living with HIV

For immunocompromised individuals living with HIV who have limited access to rapid bedside diagnostic testing, the widespread use of prophylactic antibiotics has become essential to treat and prevent infections. Cotrimoxazole is recommended long-term for people living with HIV in areas where malaria or severe bacterial infections are highly prevalent [64], due to demonstrated reductions in morbidity and mortality among HIV-positive adults and children [65–67]. The WHO also recommends cotrimoxazole for all HIV-exposed infants from 4 to 6 weeks of age until determination of the infant’s HIV-negative status [64]. Two RCTs investigated the impact on the gut microbiota of MDA with cotrimoxazole for prophylaxis in persons-living with HIV (Table 2). These RCTs were conducted in children or newborns, both in sub-Saharan Africa. One reported the impact of daily cotrimoxazole after four years of use (long-term use), and one reported the impact over the first 6 months of after birth (short-term use).

**Table 2:** Characteristics of studies included in this review of the impact of mass drug administration with antibiotics for prophylaxis in persons-living with HIV on the human gut microbiome

Reason for MDA	First author	Year	Study Design	Specimen collection and Sequencing	Country	Target population	Intervention	Major findings
HIV prophylaxis	Bourke, CD [68]	2019	RCT	150 mg stool, whole metagenome sequencing	Zimbabwe	HIV-infected children median [IQR] age 8.9 [5.7,11.1] years on Antiretroviral Therapy who were taking oral cotrimoxazole (once-daily doses of 200 mg of sulfamethoxazole and 40 mg of trimethoprim, 400 mg of sulfamethoxazole and 80 mg of trimethoprim, or 800 mg of sulfamethoxazole and 160 mg of trimethoprim for a body weight of 5 to 15, 15 to 30, or > 30 kg, respectively) (*n* = 72)	Continue versus stop daily oral cotrimoxazole	Reduced and gut-inflammation associated viridans group streptococci bacterial species abundance and gut-inflammation associated bacterial mevalonate pathway abundance after 2 years
HIV prophylaxis	D’Souza, AW [70]	2020	RCT	150 mg stool, whole metagenome sequencing	South Africa	6-week-old HIV-exposed uninfected infants (*n* = 63)	Oral cotrimoxazole (received 20 mg trimethoprim/100 mg sulfamethoxazole with < 5 kg body weight or 40 mg trimethoprim/200 mg sulfamethoxazole with 5–15 kg body weight)	Greater abundance and diversity of total genetic determinants of antibiotic resistance genes, and genetic determinants of trimethroprim (*dfr*), and sulfamethoxazole (*sul*) resistance after 6 months
HIV prophylaxis	Francis, F [69]	2020	RCT	150 mg stool, whole metagenome sequencing	Zimbabwe	HIV-infected children median [IQR] age 8.9 [5.7,11.1] years on Antiretroviral Therapy who were taking oral cotrimoxazole (once-daily doses of 200 mg of sulfamethoxazole and 40 mg of trimethoprim, 400 mg of sulfamethoxazole and 80 mg of trimethoprim, or 800 mg of sulfamethoxazole and 160 mg of trimethoprim for a body weight of 5 to 15, 15 to 30, or > 30 kg, respectively) (*n* = 72)	Continue versus stop daily oral cotrimoxazole	fivefold increase abundance of a genetic determinant of trimethoprim resistance (*dfrA1*) after 2 years

One RCT conducted in Zimbabwe, reported the impact of long-term cotrimoxazole use on the gut microbiome. HIV-positive children on ART who were taking once daily cotrimoxazole for an average of two years were randomized to continue or stop cotrimoxazole prophylaxis and followed-up for an additional two year period [67]. In accordance with the short-term 5-day course tested in children in the general population of Burkina Faso [61], long-term daily cotrimoxazole did not alter gut microbiota diversity. By contrast, cotrimoxazole suppressed gut-resident viridans group streptococci species that were associated with gut inflammation, but did not impact clinical illness, HIV progression, nutritional status, or abundance of Enterobacteriaceae [68]. These changes corresponded with reductions in systemic and gut inflammatory markers of mortality risk, providing a possible explanation for the beneficial effects of cotrimoxazole on mortality in persons living with HIV. However, continued use resulted in increased carriage of the *dfrA1* genetic determinant of resistance to trimethoprim after 2 years [69]. In contrast, other genes that confer resistance to cotrimoxazole were not affected [69].

Another RCT investigated the impact of short-term once daily cotrimoxazole use on the microbiota of HIV-exposed uninfected newborns in South Africa [70]. Consistent with long-term use in HIV-infected children in Zimbabwe, cotrimoxazole use in this HIV-exposed infant population did not significantly affect taxonomic or functional diversity [70]. However, the abundance and α-diversity of total antibiotic and cotrimoxazole resistance genes were greater in the cotrimoxazole-treated group, and these differences grew larger after 4 months of use [70]. Furthermore, there were significant differences in microbiota composition between groups as measured by β-diversity indices, which quantify the loss or gain (i.e. turnover) of bacterial taxa across an exposure gradient. Within the treated group, microbiome taxonomic, functional and resistance gene compositions became more similar over one year (i.e. reduced β-diversity), indicating that the selective pressure of cotrimoxazole shaped the microbiomes of the treated infants in a consistent way [70].

From these RCTs, cotrimoxazole does not seem to influence α-diversity but is associated with profound microbiome changes and an increase in antibiotic resistance genes during the initial period of antibiotic exposure.

#### Intrapartum antibiotic prophylaxis

Globally, the use of intrapartum antibiotic prophylaxis (IAP) is the most common strategy for preventing peripartum infections and associated adverse pregnancy outcomes [47]. IAP involves administering broad spectrum antibiotics that are effective against the microorganisms most likely to cause infections in at risk mothers, predominantly GBS [71]. IAP is recommended before incision in caesarean sections; during labor when the mother is culture positive for GBS or at risk of invasive GBS infection; or immediately after birth to reduce risk of infections associated with manual removal of the placenta, excessive intrauterine manipulations or lacerations of the genital tract [47, 48, 72, 73]. Seven observational studies and one RCT investigated the association between MDA for IAP and the gut microbiota, primarily in terms of α-diversity and β-diversity (Table 3). Three observational studies reported on infant gut microbiota composition and diversity during the neonatal period, while five observational studies and the RCT reported post-neonatal gut microbiota outcomes.

**Table 3:** Characteristics of studies included in this review of the impact of mass drug administration with antibiotics for intrapartum antibiotic prophylaxis on the human gut microbiome

Reason for MDA	First author	Year	Study Design	Specimen collection and Sequencing	Country	Target population	Intervention	Major findings
IAP	Aloisio, I [158]	2016	Cross-sectional study	200 mg stool, 16S rRNA gene V2, V3, V4, V6, V7, and V8 amplification and sequencing	Italy	Vaginally delivered, full-term neonates with no prior antibiotic exposure sampled at 6–7 days of age (*n* = 20)	2 g of IV ampicillin ≤ 4 h before delivery, then 1 g every 4 h during labor until delivery (GBS-positive mothers) versus no IAP (GBS-negative mothers)	40%–50% decreased microbiome diversity, greater abundance of Enterobacteriaceae, and reduced abundance of *Bifidobacterium* spp.
IAP	Azad, MB [76]	2016	Prospective cohort study	80–200 mg stool, 16S rRNA gene V4 amplification and sequencing	Canada	Full-term infants sampled at 3 and 12 months of age in four comparison groups: (i) no IAP with vaginal delivery, (ii) IAP with vaginal delivery, (iii) IAP with elective CS delivery, and (iv) IAP with emergency CS delivery (*n* = 198)	IAP in accordance with Canadian practice guidelines predominantly using, cefazolin (CS deliveries), IAP for GBS prophylaxis or pre-labor rupture of membranes predominantly using, penicillin (vaginal delivery)	7% decrease in microbiome diversity with vaginal delivery and IAP at 3 months, and 14% increase in microbiome diversity with CS and IAP at 1 year; greater abundance of bacteria in the Proteobacteria phylum and reduced abundance of bacteria in the Bacteroidetes phylum with IAP at 3 months and 1 year
IAP	Mazzola, G [77]	2016	Prospective cohort study	200 mg stool, 16S rRNA gene V3–V4 amplification and sequencing	Italy	Vaginally delivered, full-term infants with no prior antibiotic exposure sampled at 7 and 30 d in four comparison groups: (i) breast-fed infants born to GBS-negative mothers, not receiving IAP, (i), breast-fed infants born to GBS-positive receiving IAP, (iii) mixed-fed infants born to GBS-negative mothers not receiving IAP, (iv) mixed-fed infants born to GB positive receiving, IAP (*n* = 26)	2 g IV ampicillin at labor then 1 g every h up to a maximum of 4 g to prevent infant GBS infection (GBS-positive mothers)	Decreased microbiome diversity in breast-fed IAP exposed versus breast-fed IAP unexposed infants, greater abundance of bacteria in the Enterobacteriaceae family and reduced abundance of *Bifidobacterium* spp. in breast fed infants IAP exposed versus breast-fed IAP unexposed infants
IAP	Nogacka, A [78]	2017	Prospective cohort study	Stool, 16S rRNA gene V3 amplification and sequencing	Spain	Vaginally delivered, full-term neonates with no prior antibiotic exposure sampled at 2, 10, 30, and 90 days of age (*n* = 40)	5 million units of penicillin followed by 2.5 million units every 4 h until delivery (GBS-positive or suspected mothers) versus no IAP (GBS-negative mothers)	Reduced abundance of bacteria in the Actinobacteria phylum at 10 days and increased abundance of bacteria Firmicutes phylum at 10 and 90 days
IAP	Stearns, JC [75]	2017	Prospective cohort study	100–200 mg stool, 16S rRNA gene V3 amplification and sequencing	Canada	Vaginally delivered, full-term, singleton infants sampled at 3 d, 10 d, 6 weeks and 12 weeks (*n* = 74)	IAP with penicillin G, cefazolin, ampicillin, cephalexin to prevent infant GBS infection versus no IAP	More phylogenetically similar microbiome composition with IAP at 6 weeks of age, and reduced abundance of *Bifidobacterium* spp. and increased abundance of bacteria in the Enterobacteriaceae family at 6 and 10 weeks
IAP	Kamal, SS [79]	2019	RCT	200 mg stool, 16S rRNA gene V3-V4 amplification and sequencing	Denmark	Cesarean delivered infants sampled at 10 d and 9 months of age (*n* = 42)	Single dose of IV 1,500 mg cefuroxime administered 15–60 min prior to surgical incision versus immediately after umbilical cord clamping	15%–37% greater microbiome diversity in infants born to mothers who received IAP immediately after cord-clamping at 9 months
IAP	Coker, MO [74]	2020	Prospective cohort study	Stool, 16S rRNA gene V4–V5 amplification and sequencing	United States of America	Vaginally delivered, full-term infants sampled at 6 weeks and 12 months of age (*n* = 266)	Maternal antibiotic use for prevention of infant GBS infection categorized into the five groups: (i) no IAP, (ii) penicillin like only (amoxicillin, penicillin), (iii) cephalosporins only (cefazolin, cephalexin), (iv) multi-drug classes (two or more drugs under the category of penicillin, cephalosporin, vancomycin, clindamycin and/or gentamicin), and (v) other classes (aminoglycosides, glycopeptides or lincomycin only)	Decreased in microbiome diversity at 6 weeks with penicillin-like IAP and at 1 year with multiclass IAP; reduced abundance of *Bifidobacterium* spp. and *Bacteroides* spp. at 6 weeks and 1 year with penicillin-like IAP
IAP	Zhou, P [159]	2020	Cross-sectional study	Neonatal meconium, 16S rRNA gene V4 amplification and sequencing	China	Vaginally delivered singleton, full-term or pre-term neonates with no prior antibiotic exposure sampled at birth (*n* = 98)	2 g IV cefazolin every 12 h ≤ 48 h before delivery for the prevention of GBS newborn infection versus no IAP	Greater similarity in meconium microbiome composition among infants born to mothers who received IAP

Two cross-sectional studies and one prospective cohort study conducted in three high-income countries reported the association between IAP and gut microbiota composition during the neonatal period (Table 3). Microbiota α-diversity at 6–7 days of age was reduced in infants born to IAP-treated mothers [63]. Exposure to IAP was also associated with greater similarity in microbiota composition (i.e. reduced β-diversity), suggesting that the selective pressure of maternal antibiotic exposure had a congenerous effect on early infant microbiota composition across infants [62]. The IAP group was also dominated by Enterobacteriaceae, and depleted in *Bifidobacterium* spp. [63]. Finally, a prospective cohort study found a significant reduction in the relative abundance of the Actinobacteria phylum at 10 days of age (which includes *Bifidobacterium*) and a significant increase in the Firmicutes phylum (which includes some notable pathogenic groups, including *Staphylococcus*, *Listeria* and *Streptococcus*, as well as probiotic groups, including *Lactobacillus*) [64].

Five prospective cohort studies conducted in four high-income countries reported the association of IAP and the post-neonatal gut microbiota (Table 3). A reduction in microbiota α-diversity at age 2–3 months in IAP-exposed infants was reported in three North American cohorts [74–76]. Infants exposed to IAP had a more phylogenetically similar microbiota composition at 6 weeks of age (reduced β-diversity) as measured by UniFrac indices in one study [66], suggesting a consistent effect of maternal antibiotic exposure on the loss or gain of specific, phylogenetically-related subsets of gut bacteria. Changes in the relative abundance of bacterial taxa associated with IAP-exposure included increased Proteobacteria (a phylum that includes several enteric pathogens) [74], *Escherichia* spp. [75] and Enterobacteriaceae [77] at 1–3 months of age in three countries. IAP-exposure was also associated at age 3 months with decreased *Bifidobacterium* spp. [75, 77] in two cohorts, and an increase in the Firmicutes phylum [78]. Longer term changes included a reduction in the Bacteroidetes phylum [74, 76] and *Bifidobacterium* spp*.* at 1 year of age [74].

In spite of this evidence, it is challenging to separate the impact of maternal antibiotic exposure on the infant gut microbiota from the impact of the indications for IAP, such as cesarean delivery or GBS infection, in observational studies. One RCT tested the impact of IAP for cesarean section on infant microbiota α-diversity in Denmark (Table 3) [79]. Forty-four mothers were allocated to receive a single intravenous dose of cefuroxime either before or after umbilical cord clamping. Microbiota α-diversity was greater at 9 months in infants born to mothers who received IAP *after* cord-clamping, suggesting that later administration may ameliorate the duration of diminished infant microbiota α-diversity [79]. IAP *after* cord-clamping was also associated with greater abundance of *Bifidobacterium* spp. at 10 days. Although this difference was not statistically significant, this association may still hold biological significance since *Bifidobacterium* are essential members of the breastfed infant gut microbiota that may drive ongoing microbiota development [80] through symbiotic cross-feeding interactions with other important gut bacteria such as *Lactobacilli* [81].

Overall, these studies suggest reduced α-diversity in infant microbiota associated with maternal exposure to IAP and consistent compositional changes, including a higher relative abundance of Enterobacteriaceae and lower abundance of *Bifidobacterium*.

### Drivers of antibiotic effects on the gut microbiota

**Fig. 1:**
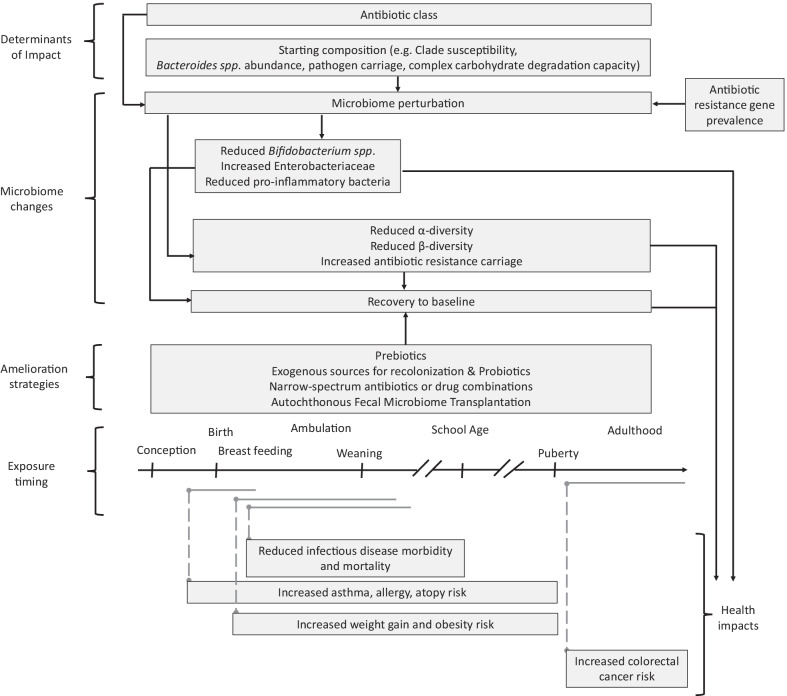
Antibiotic impacts on human gut microbiota and associated health impacts. The horizontal, black arrow represents the time axis along which key life events associated with gut microbiome development occur. Horizontal, gray, solid lines represent antibiotic exposure periods. Vertical, gray dashed lines link exposure periods to associated health outcomes. The position and length of each box relative to the time axis represents the timing and duration of microbiome changes and health effects

Recent reviews reported few studies that investigated factors which determine the impact of antibiotic exposure on microbiota composition [3, 82, 83]. However, to gain deeper insight into potential determinants of how gut microbiota respond to perturbation by antibiotics, studies that aimed to explore these factors are discussed in this section around the following common themes: antibiotic class, bacterial clades, starting microbiota composition and long-term recovery dynamics (Fig. 1).

#### Antibiotic class and bacterial clade

A recent review of 55 human studies summarized changes in microbiota composition that were significantly associated with 68 different antibiotics grouped into 22 antibiotic classes [5]. It revealed that each antibiotic most strongly affects bacteria belonging to a restricted set of genera. Furthermore, the number of affected genera varied both between and within antibiotic classes [5]. For example, azithromycin was associated with changes in the abundance of 3 genera (*Bacteroides*, *Bifidobacterium*, *Clostridium*), while other macrolides were associated with altered abundance of 2–6 genera (including the former three, as well as *Faecalibacterium*, *Prevotella*, *Ruminococcus*, *Streptococcus*, *Enterococcus*, *Haemophilus* or *Pseudomonas*). Beta-lactams, such as ampicillin and cephalosporin for example, were associated with changes in abundance of 8 and 12 genera, respectively, spanning a phylogenetically diverse range of phyla (*Actinobacteria*, *Firmicutes*, *Proteobacteria*, *Verrucomicrobia*) that differed from other beta-lactams [5].

These taxon-specific effects of different antibiotics are corroborated by culture experiments. For example, metronidazole strongly reduced the growth of *Bacteroides* spp. and *Bifidobacterium* spp. grown in anaerobic culture [84], while ampicillin significantly reduced *Bacteroides* spp. and *Clostridium perfringens*, but its effect on *Bifidobacterium* spp. depended on bacterial growth rates [84]. A more comprehensive assessment of a larger number of antibiotics tested on single species in anaerobic culture confirmed the wide ranging effects on habitual colonic bacteria [85]. For instance, aminoglycosides and sulfonamides had little effect on the bacteria tested [85]. Macrolides uniformly had bacteriostatic effects (but did not kill) all bacterial species tested, except for *Clostridium difficile*, on which they had no impact [85]. However, the effect of beta-lactams was strain specific and differed by the specific antibiotic used [85]. The impact of antibiotics on the microbiota, therefore, depends on the antibiotic used, as well as which and how bacterial clades respond.

Nevertheless, suppression of microbes that are susceptible to an antibiotic’s mechanism of action can disrupt colonization resistance and open an ecological niche for opportunistic pathogenic bacteria, increasing the host’s susceptibility to post-antibiotic infection [86, 87]. Alternatively, antibiotic use can also produce an increase in antibiotic-tolerant commensal bacteria, that have potential benefits to the host [84, 88]. Factors that influence these effects include the pharmacokinetics of different antibiotics [89, 90], the degradation and resistance mechanisms different bacteria utilize against each antibiotic [91–94], the indirect effects of these degradation and resistance mechanisms on other bacteria that do not actively perform these functions [95], and the local or regional prevalence of specific resistance determinants [96, 97]. Overall, these studies suggest that both antibiotic class and bacterial clade specificities determine the impact of antibiotics on the microbiota.

#### Starting composition of gut microbiota

Considering the variation in antibiotic effects by antibiotic class and bacterial clade, the initial composition of the microbiota may also determine the short and long-term effects of antibiotics. Analyses of data from five adult cohorts determined that the abundance of specific species with an increased functional capacity to degrade complex carbohydrates, particularly host mucins, accurately predicted microbiota recovery from antibiotic-associated reductions in α-diversity [98]. The baseline abundance of these bacteria could identify participants whose diminished α-diversity recovered after antibiotic use with 70% accuracy [98]. They proposed that capacity for mucin degradation may confer an advantage for these bacteria to repopulate the gut. Furthermore, degradation of these complex carbohydrates may produce secondary metabolites that can in turn be utilized as a nutrient source by other bacteria to support their regrowth. Recovery-associated bacteria included several species from the genus *Bacteroides*.

This finding in humans is supported by evidence from germ-free mice. Two groups were colonized with either a *Prevotella* and *Faecalibacterium* enriched or a *Bacteroides* and *Parabacteroides* enriched human fecal microbiome, and both were treated with a 7-day course of amoxicillin-clavulanate [99]. α-Diversity in the *Bacteroides* group was more resilient to antibiotic exposure after 18 days [99]. However, mucin foraging by *Bacteroides* spp. can facilitate enteric infection, which has been demonstrated for *C. difficile*, *C. rodentium*, and *S. typhimurium* in mouse models, and further community-level investigation in different age groups is required to translate the findings to human populations [86, 87, 100].

### Long-term recovery dynamics

Two comprehensive analyses provide further insights into the temporal dynamics of microbiota response and recovery from antibiotic exposure [101, 102]. Both analyses used data from adults collected longitudinally during a 10-month period that included two 5-day courses of ciprofloxacin [101, 102]. One analysis determined that the time needed for the microbiota to reach a new stable configuration varied from 2 to 42 days for different subsets of bacteria. One bacterial subset in the genus *Bacteroides* established a new equilibrium at the same or higher relative abundance within 2 weeks [102]. However, other bacteria in the *Bacteroides* genus and a subset of bacteria belonging to the family *Lachnospiraceae* increased during treatment and required 42 days to return to pre-antibiotic levels. By contrast, a group of bacteria in the family *Ruminococcaceae* was reduced to undetectable levels after a single treatment with no recovery after ten months [102]. This analysis is consistent with the aforementioned review which found bacterial clade specificities determine the impact of antibiotics on the microbiota [5]. A second analysis of the same data suggested that shorter recovery time may result from greater exposure to environmental sources of bacteria that can recolonize the perturbed microbiome [101].

Although these studies included very few individuals, and may represent inter-individual heterogeneity at best, they explored a richly sampled time-series of microbiota abundance data, and their findings are supported by experimental work. In one such experiment, two groups of mice were assigned to either specific-pathogen free conditions or general animal conditions with exposure to soil, and both were treated with a 2-week course of vancomycin and streptomycin [103]. Soil-exposed mice showed greater α-diversity and stability after treatment and recovered their pre-treatment α-diversity more quickly after antibiotic withdrawal [103]. These studies suggest that a more controlled exogenous source of bacteria, such as a complex probiotic mixture, may mitigate the impact of antibiotic exposure on the microbiome.

A third analysis used a separate dataset from a small, randomized perturbation experiment of adults to quantify the recovery time of the gut microbiota over a one-year period after exposure to different antibiotics [104]. Recovery was defined as the time from exposure to a stable microbiota configuration. The recovery time varied with the antibiotic used. The gut microbiota transitioned to a new stable configuration 4–6 months after treatment with clindamycin and ciprofloxacin [104]. By contrast, the composition of microbiota exposed to minocycline or amoxicillin was unchanged [104].

Long-term effects of antibiotic exposure have also been investigated in infants [105, 106]. Reduced α-diversity was associated with treatment immediately after birth, but recovered during the first postpartum year [106]. The reduction in α-diversity was driven by suppression of *Lachnospiraceae* and *Enterobacteriaceae* [106]. While the clinical significance of *Lachnospiraceae* is not clear, the presence of members of this family in the early gut microbiota has been associated with reduced risk of asthma [18], and *Enterobacteriaceae* are the first colonizers of the infant gut*.* Although microbiota α-diversity did largely recover by one year of age, the impact on host health of reductions in these early colonizers due to neonatal exposure to antibiotics requires careful investigation.

In another study, infants treated with antibiotics from birth to three years had lower species α-diversity compared to infants who had no antibiotic-exposure during that time, but the reduction in α-diversity was modest and was only evident for year one [105]. At the bacterial strain level, however, differences between antibiotic exposed and unexposed infants were greater [105]. Furthermore, bacterial strains were identified that only colonized the gut once. Such strains were eliminated by antibiotic-exposure but persisted in the untreated infants. By contrast, other strains were identified that recolonized the gut multiple times during follow-up. Such strains were more likely to persist in both exposed and unexposed infants, providing further support for the notion that recolonization is a driving force in microbiota recovery after antibiotic use [105].

Altogether, these analyses suggest that microbiota recovery from antibiotic-exposure may depend on the antibiotic used, the initial microbiota composition and the specific bacterial taxa affected by the drug. Furthermore, longitudinal observational studies provide additional evidence that recolonization of the gut may potentially play a critical role in microbiome recovery from antibiotic-exposure. However, more studies are needed which are designed to test the specific hypotheses suggested by these few studies.

### Associated health impacts

It is important to note that recovery alone may not protect the host from potential long-term effects of antibiotic perturbations to the microbiome (Fig. 1). Epidemiologic studies have established associations between antibiotic use and obesity; asthma, allergy, atopy; and colorectal cancer, among other health disorders. Antibiotic disruption of the gut microbiome has been proposed as an important mechanism underlying these associations [4, 83, 107]. However, most studies have investigated the relationships between antibiotic use, gut microbiome composition or function, and health outcomes separately. Recent reviews have reported little to no studies that assessed and reported antibiotic use, microbiota composition and host health outcomes in the same study population to demonstrate a direct link between antibiotic modulation of the gut microbiota and effects on health, or the specific antibiotic-induced changes to the microbiome that may be involved [4, 83, 107]. To gain further insight into the health implications of microbiome perturbation by MDA with antibiotics, I discuss such studies in this section, with reference to the findings of the MDA studies.

#### Infectious morbidity

Fifty percent or more of infants in LMICS carry at least one enteric pathogen by age 3 months and experience at least one diarrheal episode by age 24 months [108]. The most common causes of moderate-to-severe diarrhea are viral (rotavirus, sapovirus, norovirus) or bacterial (*E. coli* pathotypes, Shigella, *Campylobacter* spp.), but the prevalence of specific pathogens varies by country [109, 110]. Mortality [110] and severity [109] of diarrhea episodes also vary by country as well as by pathogen. The broad goal of MDA with antibiotics is to reduce the burden and impact of pathogenic bacteria on host health. The results of RCTs of MDA with azithromycin in LMICS suggest that suppression of *Campylobacter* spp. and pro-inflammatory microbiome metabolic pathways may partly explain the sustained reductions in child mortality due to diarrhea and dysentery observed in those settings [111–113]. Gut microbiota composition is associated with both increased and reduced severity of enteric infections [114]. Limited evidence suggest that better or worse enteric infection outcomes may vary by microbiota composition and pathogen [114]. For example, mixed diarrheagenic *E. coli* and viral infections in children were associated with increased *Bifidobacterium* spp. and more severe illness [115], but *Bifidobacterium longum* inhibited rotavirus isolates from pediatric cases and reduced the duration of viral gastroenteritis in another study [116].

Additional evidence comes from murine models. For example, mucin foraging by *Bacteroides* spp. after antibiotic perturbation of the gut microbiome can facilitate enteric infection, which has been demonstrated for *C. difficile*, *C. rodentium*, and *S. typhimurium*. [86, 87] Alternatively, gut microbiome perturbation with antibiotics has been shown to delay rotavirus infection and prevent norovirus infection in mice [117, 118]. Nevertheless, more careful elucidation of the specific microbiome components that may moderate enteric pathogen virulence is still required, particularly in human populations using well-designed, adequately powered, longitudinal studies.

Furthermore, the increase in antibiotic resistance gene carriage by the gut microbiome that can result from MDA suggests the potential for the beneficial effects to become limited by decreased susceptibility of pathogenic bacteria. Once resistance in pathogenic organisms develop, efficacy may reverse, as has been observed for other antibiotics in high-income settings [119]. In contrast, long-term use of Cotrimoxazole among children living with HIV still suppressed inflammatory viridans Streptococci after 4 years and the associated systemic inflammation that increases morality risk in this population [68]. More evidence is needed from LMICs where the availability of antibiotics [120], and treatment needs may be context specific.

#### Overweight and obesity

Antibiotic use is associated with an increased risk of obesity. The association is greater with earlier (prior to 6 months of age) and with greater frequency of exposure [121–123]. Lower gut microbiota α-diversity is also associated with obesity [14, 124]. By contrast, antibiotics can promote weight gain in undernourished children, although effects are heterogenous and are likely driven by antibiotic class as well as variable prevalence of growth-restricting comorbidities such as HIV and severe acute malnutrition in different populations [125]. Causal effects of microbiome composition on weight gain have also been demonstrated in germ free mice [13].

Without a clear understanding of the causal mechanisms that link changes in microbiota composition to health outcomes, microbiota composition is often defined as “dysbiotic” based on associations with disease relative to healthy controls [20]. In high-income adult populations, a decrease in α-diversity is a common feature of “dysbiosis” defined in terms of associations with disease. In this setting, reduced α-diversity is indicative of increased risk for obesity [126] and associated metabolic disorders such as type I [127] and II [128] diabetes. This may be particularly relevant to reduced α-diversity associated with a high-fat, low-fiber diet rich in refined-sugar [129]. By contrast, in exclusively breastfed infants the gut microbiome is dominated by *Bifidobacterium* spp. [32] due to the high concentrations of oligosaccharides present in human milk, resulting in lower α-diversity compared to mixed [32] or formula fed [32, 76] infants. Greater α-diversity is associated with earlier introduction of complementary foods [130] and increased risk of overweight in adolescence [131]. As such, greater microbiota α-diversity in early infancy may be indicative of “dysbiosis” due to inadequate early infant nutrition, thus the health implications of antibiotic-associated reductions α-diversity may be context-specific as well.

The timing of antibiotic exposure may provide further insight into context-specific effects. In a cohort of 12,422 full-term newborns in Finland, neonatal antibiotic-exposure (predominantly intravenous benzylpenicillin and gentamicin) was associated with impaired length growth among boys in the first 6 years of life. The association with growth in boys was confirmed in an independent cohort of 1707 newborns from Germany [132]. Conversely, the number of antibiotic courses received after the neonatal period, was associated with increased body mass index (BMI) at age 6 in both sexes [132]. In a subset of the Finish cohort, neonatal antibiotic-exposure was associated with decreased abundance of the genus *Bifidobacterium*, as well as reduced α-diversity at 1 month, but greater α-diversity beyond age 12 months [132]. Notwithstanding the later increase in overall α-diversity, diversity within the *Bifidobacterium* genus remained decreased at 24 months [132].

In the same study, when germ-free mice were colonized with fecal microbiomes from 1-month old antibiotic-exposed infants, male mice experienced reduced weight gain compared to mice colonized with an antibiotic-unexposed infant microbiome. However, female mice did not, corroborating the observational findings in the human cohorts [132]. The results of this study show that the timing of antibiotic exposure in infancy, and sex-related host differences, can have a profound impact on the early microbiota and infant development.

While a causal effect of early-life antibiotic exposure on obesity is yet to be confirmed [122], and the mechanism is unclear, some *Bacteroides* spp. associated with microbiota recovery (*B. caccae, B. intestinalis, B. uniformis*) are also associated with reduced BMI. This may be due to their increased capacity to degrade complex carbohydrates [98]. By contrast, upregulation of microbiome metabolic pathways involved in biosynthesis of simpler sugars via degradation of more complex, host-derived polysaccharides is also associated with increased BMI and insulin resistance [133]. Forty percent of the BMI-associated and insulin resistance-associated sugar metabolism pathways in this study were upregulated after beta-lactam treatment. [133]

A recent study in mice demonstrated that low-dose penicillin delivered from birth elicited an increase in adiposity and exacerbated the effect of a high fat diet on increased body weight. The growth phenotype was induced in germ-free inoculated with fecal microbiota from antibiotic treated mice, showing that the altered microbiota plays a causal role [134]. Antibiotic exposed gut microbiota were characterized by reduced abundance of *Lactobacillus* spp., *Allobaculum* spp., *Rikenellaceae* spp., and *Candidatus arthromitus* [134]. The increased adiposity induced by neonatal antibiotic exposure in these mice is consistent with the increased BMI associated with post-neonatal exposure previously described [132]. However, more research is needed to characterize and confirm the components of the microbiome that promote recovery from antibiotic exposure, those that may be required for healthy metabolism, and to translate these mechanism to human health.

#### Asthma and atopy

Earlier [135, 136] and more frequent antibiotic prescription is also associated with increased risk of childhood asthma [137–139]. Lower gut microbiota α-diversity is associated with eczema and allergic sensitization in infancy and childhood as well [18, 140, 141]. A direct link between microbiota alterations by infant antibiotic use and asthma risk at age 5 years was recently demonstrated [142]. Infants exposed to antibiotics in the first year of life were two times as likely to develop asthma by age 5. An estimated 25% of this association was attributable to antibiotic-induced reductions in α-diversity or antibiotic-associated changes in the abundance of specific taxa [142]. Although there is tremendous variability in the gut microbiome through infancy, a meaningful fraction of the infancy microbiome is acquired just after birth from the maternal gut [31] and is retained up to 3 years [127]. Maternal exposure to antibiotics, such as with IAP, may, therefore, also alter microbial species transmission to the infant during delivery and impact early colonization. Antibiotic exposures that impact neonatal microbiome composition may have long-term consequences for child development. Several studies using animal models have shown that the neonatal period is critical for immune system maturation, a process that is dependent on microbiome colonization of the neonatal gut [18, 138, 139, 143–145] and disrupted by antibiotic ablation of the gut microbiome [143, 145].

### Promising preventive measures to protect the microbiota

A few promising approaches have been suggested to mitigate the impacts of antibiotic use on the microbiome (Fig. 1). Co-administration of prebiotics to promote the growth of commensal bacteria is one such strategy. In infants, human milk is rich in complex oligosaccharides that serve as substrates for the growth of *Bifidobacterium spp*. and facilitate cross-feeding by other species [81]. Breastfeeding is associated with quicker recovery of microbiome α-diversity in IAP-exposed infants in one observational cohort [77]. The benefits of breastfeeding may also extend beyond its prebiotic content [146]. In children, co-administration of lactulose with azithromycin helped to restore the relative abundance of *Lactobacillus*, *Enterococcus*, *Anaerostipes*, *Blautia* and *Roseburia* within 18 days of treatment, while azithromycin alone caused an increase in the abundance of proinflammatory *Streptococci* up to 60 days after treatment [147].

Co-administration of probiotics with antibiotics in adults has also shown some potential to mitigate antibiotic selection for genetic determinants of resistance [148]. However, successful gut colonization by orally administered probiotic bacteria has shown considerable variability by person, gut-region and probiotic strain, explained by host and autochthonous microbiome features [149]. Also, absence of resistance to the administered antibiotic among the probiotic strains could exacerbate selection for antibiotic resistance in the microbiome [150], further limiting the potential protective effects of probiotics. Fecal microbiota transplantation using a self-provided, healthy fecal specimen collected prior to antibiotic administration may be a more effective approach to microbiome restoration [151], although this may be a less practical option for LMICs.

Another strategy involves use of antibiotic-drug combinations to achieve more targeted species-specific effects than single antibiotic treatments. For example, one investigation screened > 1000 drugs to identify candidates that reduce the broad-spectrum activity of antibiotics without impairing their activity against relevant pathogens [85]. The anticoagulant drug dicumarol, and two non-steroidal anti-inflammatory drugs, tolfenamic acid and diflunisal, emerged as strong inhibitors of the effect of erythromycin on commensal gut bacteria (e.g. *Bacteroides vulgatus* and *Bacteroides uniformis*). However, erythromycin’s impact on pathogenic bacteria (e.g. *Staphylococcus aureus*, *Streptococcus pneumoniae* and *Enterococcus faecium*) was not impacted [85]. Another investigation profiled almost 3,000 combinations of antibiotics, drugs, and food additives to identify candidate compounds that could mitigate the collateral impacts on the microbiome without reducing the effect on the pathogens (e.g. *Escherichia coli*, *Salmonella enterica serovar Typhimurium* and *Pseudomonas aeruginosa*) [152]. More than 70% of the drug combinations had narrow species-specific effects and 20% showed strain-specific effects [152].

Identifying narrow-spectrum antibiotic alternatives is another approach. The microbiome itself may be a source of such narrow-spectrum compounds. For example, investigation of 752 bacterial genomes from the human microbiome project identified a cluster of genes carried by commensal bacteria that encode thiopeptides [153]. Lactocillin, a thiopeptide encoded by *Lactobacillus gasseri* showed strong inhibitory activity against common pathogens such as *Streptococcus aureus* and *Gardnerella vaginalis*, but not against commensals [153]. Thuricin-CD is another such antimicrobial that has been identified, which is produced by *Bacillus thuringiensis* [154]. It has been shown to be effective against *Clostridium difficile* without impacting microbiota composition in a fecal-culture system that models the human colon [154]. Development of such interventions will require deeper understanding of the mechanisms by which MDA with antibiotics produce health benefits, or undesirable side-effects, so that narrow-spectrum alternatives can achieve the desired outcomes while minimizing unwanted risks.

Finally, there may also be important microbial components in the environment that may help to prevent dysbiosis or restore physiologically important subsets of the gut microbiome during critical developmental periods [155, 156]. Identification of such components could guide development and testing of mitigating interventions [157].

### Limitations

This is a narrative review restricted to studies of antibiotics given as part of a mass drug administration program or policy to reduce morbidity and mortality in specific at-risk target populations, rather than treatment for a specific infection. Due to current public health interest in MDA with antibiotics to reduce childhood morbidity and mortality in view of global targets, and longer-standing interest in trachoma and yaws elimination programs, I focused on antibiotics administered in these contexts. I also focused on antibiotic prophylaxis in persons-living with HIV and intrapartum antibiotic prophylaxis due to the existence of recommendations and guidelines for those uses. There are few such publications and they are limited to infants, children, and pregnant women, reducing generalizability to populations outside these risk groups or to other indications for treatment. Most of these studies only reported antibiotic impacts on α- and β-diversity metrics, precluding more in-depth analysis of antibiotic-associated taxonomic changes. To gain further insight into the results of these MDA studies, I also discussed studies that reported the factors which determine how gut microbiota respond to antibiotic perturbation and studies which investigated antibiotic use, gut microbiota, and health outcomes in the same study population. The number of such studies was also small, limiting the conclusions that can be drawn. Finally, most studies reviewed here used 16S amplicon sequencing, and did not provide any information regarding the functional capability of the microbiome, which could provide more insight into the effects of antibiotic-induced alterations on health and the mechanisms involved.

## Conclusions

The most consistent reported impact of MDA with antibiotics has been a reduction in microbiota α-diversity in infants and children, in addition to a reduction in *Bifidobacterium* spp. and an increase in Enterobacteriaceae with prenatal or neonatal exposure. The microbiome may not fully return to its initial composition, depending on the class of antibiotic used, the initial composition of the microbiome, or the sources and frequency of recolonization. The potential impact on host health may depend on the timing of antibiotic exposure and the specific context. Interventions to mitigate or monitor the impacts of antibiotic use on the microbiome show promise but require further testing. Microbiome alteration by antibiotics is a complex process, and more comprehensive studies are required to fully characterize the potential mechanisms of antibiotic-induced changes in the human gut microbiome and consequences for host health, and to inform their translation into alternative treatments and public health strategies.

## Data Availability

All data generated or analyzed during this study are included in this published article.

## Abbreviations

ART: Antiretroviral Therapy
BMI: Body Mass Index
DNA: Deoxyribonucleic acid
GBS: Group B Streptococcus
HIV: Human Immunodeficiency Virus
IAP: Intrapartum Antibiotic Prophylaxis
LMIC: Low- and Middle-Income Country
MDA: Mass Drug Administration
PCR: Polymerase Chain Reaction
RCT: Randomized Controlled Trial
RNA: Ribonucleic acid
rRNA: Ribosomal Ribonucleic acid
WGS: Whole Genome Sequencing
WHO: World Health Organization
